# CXCL9 and CXCL10 support the exacerbated humoral response in recovered COVID-19 patients who developed acute respiratory distress syndrome by promoting plasma cell differentiation, whereas CXCL9 also induces CD40L and CXCR3 upregulation on T helper cells

**DOI:** 10.3389/fimmu.2025.1684704

**Published:** 2025-12-04

**Authors:** Romina Quiroga, Sergio Sanhueza, Catalina Sepúlveda, Bárbara Antilef, Camila Muñoz, Camilo Cabrera, Marco Fraga, Faryd Llerena, Liliana Lamperti, María Inés Barría, Alicia Colombo, Gonzalo Labarca, Mario Henríquez-Beltrán, Luciano Ferrada, Estefanía Nova-Lamperti

**Affiliations:** 1 Molecular and Translational Immunology Laboratory, Department of Clinical Biochemistry and Immunology, Pharmacy Faculty, University of Concepcion, Concepcion, Chile; 2 Facultad de Odontología, Universidad San Sebastián, Concepción, Chile; 3 Facultad de Medicina, Universidad San Sebastián, Puerto Montt, Chile; 4 Basic and Clinical Oncology Department, Faculty of Medicine, University of Chile, Santiago, Chile; 5 Pathological Anatomy Service, Clinical Hospital at University of Chile, Santiago, Chile; 6 Department of Pathological Anatomy, Faculty of Medicine, University of Chile, Santiago, Chile; 7 Internal Medicine, Complejo Asistencial Dr. Víctor Ríos Ruiz, Los Ángeles, Chile; 8 Division of Sleep and Circadian Disorders, Brigham and Women’s Hospital, Harvard Medical School, Boston, MA, United States; 9 Translational Research in Respiratory Medicine, Biomedical Research Institute of Lleida (IRBLleida), Hospital Universitari Arnau de Vilanova-Santa Maria, Lleida, Spain; 10 Escuela de Kinesiología, Facultad de Salud, Universidad Santo Tomás, Los Ángeles, Chile; 11 Núcleo de Investigación en Ciencias de la Salud, Universidad Adventista de Chile, Chillán, Chile; 12 Centro de Investigación Biomédica en Red (CIBER) of Respiratory Diseases (CIBERES), Institute of Health Carlos III, Madrid, Spain; 13 Centro de Microscopía Avanzada Biobío (CMA), University of Concepcion, Concepcion, Chile

**Keywords:** COVID-19, IgG, antibodies, B cells, plasmablasts, chemokines, CXCL9, CXCL10

## Abstract

**Background:**

Severe COVID-19 is frequently associated with acute respiratory distress syndrome (ARDS) and prolonged pulmonary sequelae. Persistent immune activation, including dysregulated B cell responses and increased proinflammatory chemokines, has been linked to the post-acute sequelae of SARS-CoV-2 infection. However, the mechanisms linking these factors remain poorly defined.

**Methods:**

Sixty patients were studied four months after acute COVID-19, including 34 who developed ARDS, 26 who did not develop ARDS, and 12 healthy controls. Clinical, computed tomography scan (CT), and diffusion capacity of the lungs for carbon monoxide (DLCOc) assessments were performed. Anti-SARS-CoV-2 IgM/IgG levels were quantified, circulating B cell subsets were characterized, and circulating cytokines and chemokines were measured. CXCR3 expression on B cells was analyzed by spectral flow cytometry. *In vitro* assays were performed to evaluate the effects of CXCL9 and CXCL10 on B cell activation, plasma cell differentiation, IgG production, and CD40L expression on CD4^+^ T cells. Associations between immunological markers and pulmonary sequelae were assessed.

**Results:**

IgG, but not IgM, levels were significantly higher in patients with ARDS than in patients without ARDS. Both COVID-19 groups showed a reduction in CD19^+^CD20^+^ B cells and an increase in plasmablasts compared to controls. Serum levels of CXCL9 and CXCL10, but not other cytokines, positively correlated with IgG levels. *In vitro*, CXCL9 increased CD86 expression on B cells, while both chemokines promoted plasma cell differentiation (CD27^+^CD38^+^, CD138^+^) and increased total IgG secretion. CXCL9 also increased the expression of CXCR3 and CD40L on activated CD4^+^ T cells. Clinically, patients with combined CT abnormalities and reduced DLCO had the highest levels of IgG, CXCL9, and CXCL10.

**Conclusion:**

Four months after COVID-19, patients with prior ARDS and persistent pulmonary sequelae exhibit sustained elevations of anti-SARS-CoV-2 IgG and chemokines CXCL9 and CXCL10. Both chemokines directly enhance B cell differentiation into IgG-secreting plasma cells *in vitro*, while CXCL9 also increases CD4^+^ T cell help, suggesting a mechanistic link between chronic inflammation, increased humoral responses, and long-term lung impairment. Targeting CXCL9/CXCL10–CXCR3 signaling could offer therapeutic potential to mitigate post-COVID pulmonary complications.

## Graphical Abstract

**Figure:**
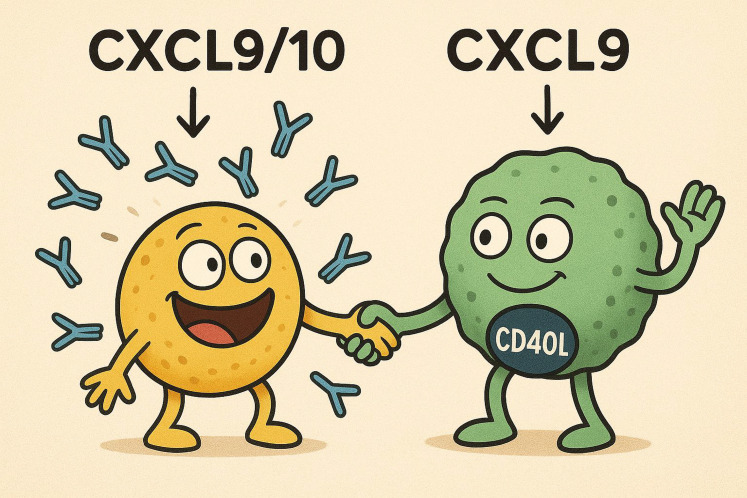

## 1 Introduction

The emergence of the coronavirus disease 2019 (COVID-19) pandemic, caused by the SARS-CoV-2 virus, had a significant global impact, challenging worldwide health systems and boosting the development of biotechnological applications and new scientific research lines (1). In this pathology, most cases present mild disease, with symptoms such as fever and cough, while moderate cases are characterized by dyspnea and low oxygen saturation (2, 3). In a subset of patients, COVID-19 progresses to acute respiratory distress syndrome (ARDS), characterized by severe systemic inflammation and significant respiratory function impairment, potentially leading to pulmonary failure, septic shock, and multi-organ failure, which increases the risk of death (4, 5). This group of patients develops a phenomenon known as a cytokine storm, characterized by abnormal activation of immune cells and the release of excessive pro-inflammatory cytokines and chemokines. These include interferon-gamma (IFN-γ), interleukins such as IL-1β, IL-6, IL-12, tumor necrosis factor-alpha (TNF-α), transforming growth factor-beta (TGF-β), and chemokines such as CXCL10, CXCL8, CXCL9, CCL2, CCL3, and CCL5 (6, 7). The exacerbation of these mediators has been previously associated with several causes, including viral and bacterial infections, chronic inflammation, cancer metastasis, certain immunotherapies, and autoimmune diseases such as rheumatoid arthritis (8–13).

In addition to the secretion of inflammatory cytokines, antibody production represents a fundamental mechanism for viral elimination. In the context of COVID-19, the dynamics and regulation of the humoral immune response—particularly in severe disease—remain incompletely understood. Notably, studies have shown that patients with severe forms of the disease exhibit higher levels of anti-SARS-CoV-2 IgG antibodies compared to those with mild or moderate illness. However, the factors contributing to this differential antibody response remain poorly understood and have yet to be thoroughly investigated (14–16). These findings suggest that these patients experience an exacerbated humoral response, in which the antibody production process is likely being modulated or enhanced at some stage.

Antibody production is dependent on appropriate B cell activation and differentiation, a process that can occur in a T cell-dependent or -independent manner. In T cell-dependent responses, the interplay between CD40 ligand (CD40L) on helper T cells and CD40 on B cells is essential, along with a coordinated signaling milieu involving diverse cytokines and chemokines responsible for B cell activation, migration, and phenotypic maturation (17, 18). Despite this fundamental knowledge, it remains unclear at what stage of this complex differentiation process antibody production is exacerbated in severe COVID-19 cases, nor which are the key immunological factors driving this response.

The aim of this research was to characterize B cell subsets and the humoral immune response in a cohort of recovered patients who had different degrees of COVID-19 severity, as well as different systemic factors that mediate the humoral response. We observed that COVID-19 patients who developed acute respiratory distress syndrome (ARDS) exhibited significantly higher levels of anti-SARS-CoV-2 IgG compared to COVID-19 patients without ARDS. When correlating antibodies levels with systemic inflammatory factors 4 months after the acute phase of the disease, we found that CXCL9 and CXCL10 were the main chemokines associated with IgG secretion. To determine whether these chemokines contributed to the exacerbation of the humoral response in severe patients, we evaluated a plasma cell activation and differentiation protocol in the presence or absence of CXCL9 and CXCL10. Our results revealed that CXCL9 and CXCL10 significantly increased the percentage of CD38^hi^CD27^hi^ cells, CD138^+^ cells and IgG secretion. CXCL9 also increased CD86 expression in B cells and CD40L and CXCR3 upregulation in CD4^+^ T cells. These findings proposed CXCL9 and CXCL10 as two chemokines supporting the humoral response in severe COVID-19 and in other pathologies where the release of these chemokines is exacerbated.

## 2 Materials and methods

### 2.1 Patient enrollment

Sixty patients confirmed with SARS-CoV-2 infection through positive PCR tests between the months of April to July 2020 from Dr. Víctor Ríos Ruiz Hospital were included in the study. Patients with prior respiratory comorbidities (asthma, chronic obstructive pulmonary disease, or other respiratory conditions), those over 70 years old, participants with missing follow-up, history of transfer to another hospital or city post-discharge, individuals in palliative care, or those with mental disabilities hindering assessments were excluded. Patients were categorized based on the presence or absence of Acute Respiratory Distress Syndrome (ARDS) during COVID-19 according to the Berlin criteria (19). Patient samples were taken at 4 months post-infection between the months of August to November 2020. Beside ARDS during acute infection, patients were categorized based on lung sequelae at 4 months post-infection, by analyzing the presence of structural lung sequelae determined by a chest computed tomography scans or functional lung sequelae determined by the DLCO score (Diffusion capacity of carbon monoxide test). Additionally, 12 healthy donors without confirmed COVID-19, with weekly negative PCR test during the same 4 months and in the absence of anti-SARS-CoV-2 antibodies at the time of sampling were included. The study was approved by the scientific ethical committee (CEC) of the Bio Bío Health Service (code: CEC113). Patients were recruited following STROBE treaty guidelines. At the time of enrollment, signed informed consent was obtained, and all methods were conducted following the Helsinki Declaration and good clinical practices.

### 2.2 Lung function and structure tests

Pulmonary tests were assessed as previously reported by our research group (20). To evaluate pulmonary function, diffusing capacity for carbon monoxide (DLCO) was performed. DLCO (Elite PlatinumDL; Medical Graphics Inc., USA) was corrected using barometric pressure: hemoglobin (DLCOc), % ml/min/mmHg, DLCOc 80%, alveolar volume (AV, %) and DLCO/AV ratio (%). A DLCOc <80% was considered abnormal. Computed tomography was used to evaluate lung structure. All images were acquired using a high-resolution computed tomography scanner (SOMATOM, Siemens, Germany). Imaging and grading (normal or abnormal chest CT) were defined by a radiologist blinded to medical records, reporting: ground-glass opacities, mixed ground-glass opacities, consolidation, interlobular thickening, bronchiectasis, atelectasis, solid nodules, nonsolid nodules, reticular lesions, fibrotic lesions, air trapping, and the number of affected lobes. The total severity score (TSS) was used to quantify chest CT abnormalities based on visual inspection of each lobe, reporting the % impairment for each lobe (0–25%: 1 point; 26–50%: 2 points, 51–75%: 3 points, and 76–100%: 4 points), with the sum for each lobe representing the TSS. A TSS >1 was considered an abnormal CT.

### 2.3 Serum sample collection

Blood was collected in tubes with separating gel at 4 months post-infection. Samples were centrifuged at 3500 rpm for 12 minutes for serum separation. Serum was immediately used to measure IgM and IgG antibodies.

### 2.4 Determination of IgM and IgG Anti-SARS-CoV-2 antibody levels

An immunoenzymatic chemiluminescence assay was conducted using the MAGLUMI^®^ 2019-nCoV IgM kit (SNIBE) to determine IgM antibody levels. Similarly, an indirect immunoenzymatic chemiluminescence assay was performed using the MAGLUMI^®^ 2019-nCoV IgG kit (SNIBE) to determine IgG antibody levels. Both determinations were automatically processed using the MAGLUMI 800 analyzer for measurement.

### 2.5 Determination of cytokine and chemokine levels

Pro-inflammatory cytokines (IL-12, IL-1β, IL-6, IL-8, and TNF-α) and chemokines (CCL5, CCL2, CXCL9, and CXCL10) were quantified using the BD Cytometric Bead Array (CBA) Human Inflammatory Cytokines Kit (Cat. No. 551811, BD Biosciences) and the BD CBA Human Chemokine Kit (Cat. No. 552990, BD Biosciences), respectively. Data acquisition was performed on an LSRFortessa™ X-20 flow cytometer (BD Biosciences) and analyzed by FCAP Array Software v3.0 (BD Biosciences).

### 2.6 Isolation of peripheral blood mononuclear cell from peripheral blood

Blood was collected by venipuncture into collection tubes with EDTA. Mononuclear cells from peripheral blood of healthy donors and patients were isolated using a density gradient separation with Ficoll-Paque™ PLUS (Cytiva, Cat. Number 17144002). The isolated cells were washed with Phosphate Buffered Saline (PBS) buffer (Gibco, Cat. Number 18912-014). Cell count was determined using trypan blue reagent as an indicator of cell death using a Neubauer chamber. An aliquot of peripheral blood mononuclear cell (PBMC) was cryopreserved at -80 °C for further analysis, while fresh cells were used to analyze B cell populations.

### 2.7 Analysis of B cell populations

Peripheral blood mononuclear cell (PBMC) samples from healthy donors and patients were analyzed by flow cytometry to evaluate the expression of the following markers: CD19-APC-eFluor 780 (eBioscience, Cat. No. 47-0199-42, clone HIB19), CD20-eFluor 5780 (eBioscience, Cat. No. 56-0209-42, clone 2H7), CD27-APC (eBioscience, Cat. No. 17-0279-42, clone O323), CD38-PE-Cy7 (BioLegend, Cat. No. 303516, clone HIT2), CD24-PE (eBioscience, Cat. No. 12-0247-42, clone eBioSN3), IgM-PerCP-Cy5.5 (BD Pharmingen™, Cat. No. 561285, clone G20-127), and IgD-PE (BD Pharmingen™, Cat. No. 555779, clone IA6-2). Flow cytometric analysis was performed using a BD Fortessa X20 cytometer, and data was processed and analyzed with FlowJo software (BD).

### 2.8 Analysis of Anti-SARS-CoV-2 B Cells (RBD B cells)

A total of 34 cryopreserved PBMC samples stored at –80 °C were used for analysis. Among these, 21 samples were from patients who developed ARDS and 13 from patients without ARDS. The detection of receptor-binding domain (RBD)-specific B cells was performed using the SARS-CoV-2 RBD B Cell Analysis Kit, anti-human (Miltenyi Biotec), following the manufacturer’s instructions. Briefly, the kit enables identification of RBD-specific B cells through the formation of RBD tetramers. These tetramers are generated by conjugating a recombinant SARS-CoV-2 RBD protein to biotin and streptavidin, followed by labeling with either PE or PE-Vio 770 fluorophores. B cells capable of simultaneously binding both fluorophore-conjugated tetramers were identified as RBD-specific B cells.

### 2.9 Isolation of B and T Cells from peripheral blood of healthy donors for functional assays

Blood samples from healthy donors were used in functional assays. From the isolated mononuclear cells, B and T cells were separated using Magnetic-activated cell sorting (MACS). Initially, positive selection for the CD4 antigen was performed. Subsequently, negative selection for B cells (CD20+) was carried out with the remaining CD4^-^ cells. Both separations were conducted using separation kits, following protocols provided by Miltenyi Biotec (CD4+ T Cell Isolation Kit, human; Cat. Number 130-096-533; B cell Isolation Kit II, human; Cat. Number 130-091-151). Finally, the purity of both cell populations was analyzed by evaluating CD20 and CD4 using flow cytometry.

### 2.10 Three-phase culture system for activation and differentiation of B cells

An optimized three-phase culture system was developed with CD20+ B cells isolated from peripheral blood of healthy donors. Were cultured in RPMI-1640 medium (Cytiva; Cat. Number SH30255.2) supplemented with fetal bovine serum (FBS) (Cytiva; Cat. Number SH30068.03HI) at 10% and IL-2 (500 U/mL) (Novartis Pharmaceuticals UK Limited; Cat. Number PL-00101/0936) with Penicillin/Streptomycin antibiotics (Gibco; Cat. Number 10378-016) in a 96-well round- bottom plate (U-bottom). The cell density used was 1.5 × 10^5 cells in 200 uL of culture medium per well. Phase I involved IL-2 (20 U/mL; Proleukin Novartis), IL-10 (50 ng/mL; BioLegend Cat. Number 571004), IL-15 (10 ng/mL; BioLegend, Cat Number 570304), IL-21 (50 ng/mL; BioLegend, Cat. Number 571202), Oligodeoxynucleotide CpG 2006 (10 μg/mL; InvivoGen, Cat. Number tlrl-2006-1), Recombinant RBD Antigen (10 μg/mL; BioLegend Cat. Number 793604), and Mega CD40L (0.5 μg/mL; Enzo, Cat. Number ALX-522-110-C010). Cells were cultured for 4 days. Phase II used IL-2 (20 U/mL), IL-10 (50 ng/mL), IL-15 (10 ng/mL), and IL-21 (50 ng/mL) for 3 days. Finally, in Phase III, IL-15 (10 ng/mL), IL-6 (50 ng/mL; BioLegend, Cat Number 570804), Type I IFN (50 ng/mL; R&D Systems, Cat. Number 11020-IF), and B cell activating factor (BAFF) (100 ng/mL; BioLegend, Cat. Number 559602) were used for 3 days.

### 2.11 Three-phase culture system with addition of CXCL9 and CXCL10 chemokines

To assess the impact of CXCL9 and CXCL10 on B cell cultures, these chemokines were incorporated into the activation process during phases II and III of culture. A concentration of 1 ng/mL CXCL9 (BioLegend, Cat. Number 578102) and 8 ng/mL CXCL10 (BioLegend, Cat. Number 573502) was used.

### 2.12 Phenotypic evaluation of B Cell Activation and differentiation protocol

B cell activation and differentiation were assessed by flow cytometry at the end of each experimental phase. Dead cells were excluded from the analysis using Ghost Dye™ (Alexa780; TONBO Biosciences Cat. Number 13-0865-T100). Two antibody panels were employed: one to detect activation markers (CD86-PE (BioLegend, Cat. Number 374206, clone BU63), CD25-PECy7 (BioLegend, Cat. Number 356108, clone M-A251) and HLA-DR-APC (BioLegend, Cat. Number 308622, clone L243)) and another to identify differentiation markers (CD27-APC, CD38-PECy7, and CD138-FITC (BioLegend, Cat Number 352304, clone DL-101)). Also, an anti-CXCR3-FITC antibody (BioLegend; Catalog No.: 353704) was used for chemokine receptor expression.

### 2.13 *In vitro* Total IgG antibody production analysis (ELISA)

Total IgG antibody levels were quantified in the culture supernatant of the phase III using the RayBio^®^ Human IgG ELISA Kit (Cat. Number ELH-IGG 0612230225).

### 2.14 CXCR3 Receptor expression on B Cells

Basal expression of CXCR3 was determined in B cells from healthy donors on a panel of markers including anti-CXCR3-FITC, CD27-APC, CD38-PECy7, IgM-PerCP-Cy5.5, IgD-PE and IgG-VioBlue (Miltenyi, Cat. Number 130-128-032, clone IS11-3B2.2.3) by Spectral Flow Cytometry using Cytek^®^ Aurora.

### 2.15 Determination of CXCL9 and CXCL10 effects on CD40L expression in *in vitro* activation of T helper cells

CD4^+^ T cells were stimulated with anti-CD3 and anti-CD28 activation beads (Gibco; Catalog No.: 11131D) in RPMI-1640 medium supplemented with 10% FBS and IL-2 (500 U/mL). Cells (1.5 x 10^5 cells/well) were cultured in 96-well round-bottom plates under three conditions: activated control, activated with CXCL9 (1 ng/mL), or activated with CXCL10 (8 ng/mL). CD40L-PE (BioLegend, Cat. Number 310805, clone 24-31), CD25-PECy7, and CXCR3-FITC expression were assessed by flow cytometry at baseline and 6, 12, 24, and 48-hours post-activation. Ghost Dye™ for viability exclusion was used.

### 2.16 Statistical analysis

Data analysis was performed using GraphPad Prism 9. Data were analyzed using *t*-tests, Wilcoxon test, repeated measures one-way ANOVA, repeated measures two-way ANOVA or Kruskal-Wallis test where appropriate. Significance was depicted as * (P < 0.05), ** (P < 0.01), *** (P < 0.001), or **** (P < 0.0001).

## 3 Results

### 3.1 COVID-19 patients with ARDS exhibit a higher presence of anti SARS-CoV-2 IgG antibodies at 4 months post-infection

To study the antibody response against SARS-CoV-2, 60 patients with COVID-19 were recruited, from which 34 patients developed ARDS during the acute phase of the infection (**Table 1**). In addition, 12 healthy donors without COVID-19 were invited to participate in the study as a control group (**Supplementary Table S1**). Four months after acute COVID-19, blood and serum samples were collected. Patients were evaluated by measuring clinical biochemistry, inflammatory parameters, SARS-CoV-2-specific IgM/IgG levels, and cell population studies. In addition, medical examinations and functional tests such as CT scans and DLCOc examinations were performed to characterize lung dysfunction. Patients with abnormal CT scan [defined as the total severity score (TSS) >1] and abnormal DLCOc examination adjusted by hemoglobin (defined as DLCOc <80%) were identified as patients with pulmonary sequelae as previously described by us (20).

**Table 1:** Description of the clinical characteristics in the study cohort (n=60).

Cohort characteristics	ARDS	No ARDS	p-value
Gender Male: Female (%)	23:11 (67.6:32.4)	9:17 (34.6:65.4)	0,0110*
Age (years), (SD)	52,0 ± 11,7	40,4 ± 12,7	n.s.
ABO Group			n.s.
A, *N* (%)	8 (23,5)	6 (23,1)	n.s.
B, *N* (%)	4 (11,8)	2 (7,7)	n.s.
AB, *N* (%)	2 (5,9)	0 (0)	n.s.
O, *N* (%)	20 (58,8)	18 (69,2)	n.s.
Measurements			
Weight, Kg (SD)	87,9 ± 15,9	79,2 ± 13,4	n.s.
Height, cm (SD)	165,1 ± 9,4	163,7 ± 9,6	n.s.
BMI, Kg/m^2^ (SD)	31,8 ± 5,1	29,2 ± 4,5	n.s.
Neck circumference, cm (SD)	43,3 ± 4,9	40,0 ± 5,0	n.s.
Waist circumference, cm (SD)	107,8 ± 12,2	98,1 ± 11,6	n.s.
Hip circumference, cm (SD)	110,2 ± 9,9	106,4 ± 8,6	n.s.
Tabaco status			n.s.
Current, *N* (%)	3 (8,8)	5 (19,2)	n.s.
Former, *N* (%)	11 (32,4)	4 (15,4)	n.s.
Never smoker, *N* (%)	20 (58,8)	17 (65,4)	n.s.
Alcohol usage			n.s.
Never, *N* (%)	14 (41,2)	11 (42,3)	n.s.
Occasionally, *N* (%)	18 (52,9)	15 (57,7)	n.s.
Frequently, *N* (%)	2 (5,9)	0 (0)	n.s.
Symptoms during acute phase			
Fever, *N* (%)	22 (64,7)	14 (38,9)	n.s.
Headache, *N* (%)	17 (50,0)	20 (76,9)	0,0335*
Chest pain, *N* (%)	16 (47,1)	11 (42,3)	n.s.
Sore throat, *N* (%)	14 (41,2)	12 (46,2)	n.s.
Cought, *N* (%)	25 (73,5)	14 (53,9)	n.s.
Dyspnea, *N* (%)	31 (91,18)	13 (50,0)	0,0004***
Polypnea, *N* (%)	27 (79,4)	9 (34,6)	0,0004***
Myalgia, *N* (%)	19 (55,9)	20 (76,9)	n.s.
Desaturation, *N* (%)	2 (5,9)	1 (3,9)	n.s.
Abdominal pain, *N* (%)	8 (23,5)	11 (42,3)	n.s.
Diarrhea, *N* (%)	9 (26,5)	12 (46,2)	n.s.
Change smell, *N* (%)	13 (38,2)	12 (46,2)	n.s.
Change taste, *N* (%)	11 (32,4)	13 (50,0)	n.s.
Comorbidities			
Arterial hypertension, *N* (%)	14 (41,2)	6 (23,1)	n.s.
IR at baseline, *N* (%)	10 (29,4)	1 (3,9)	0,0164*
T2DM at baseline, *N* (%)	3 (8,8)	4 (15.4)	n.s.
Heart failure, *N* (%)	0 (0)	0 (0)	
COPD, *N* (%)	0 (0)	0 (0)	
Previous cancer, *N* (%)	1 (2,9)	0 (0)	n.s.
CKD, *N* (%)	0 (0)	0 (0)	
Afib, *N* (%)	0 (0)	1 (3,9)	n.s.
Stroke, *N* (%)	1 (2,9)	0 (0)	n.s.
CHD, *N* (%)	0 (0)	0 (0)	
NAFLD, *N* (%)	5 (14,7)	2 (7,7)	n.s.
Hypothyroidism, *N* (%)	4 (11,8)	1 (3,9)	n.s.
4-months after-COVID-19			
Pulmonary tests			
Abnormal CT, *N* (%)	30 (88,2)	7 (26,9)	****<0,0001
DLCOc <80%, *N* (%)	12 (35,3)	7 (26,9)	n.s.
Symtoms			
Fever, *N* (%)	0 (0)	0 (0)	
Headache, *N* (%)	9 (26,5)	12 (46,2)	n.s.
Chest pain, *N* (%)	3 (8,8)	1 (3,9)	n.s.
Sore throat, *N* (%)	4 (11,8)	1 (3,9)	n.s.
Cough, *N* (%)	6 (17,7)	6 (23,1)	n.s.
Dyspnea, *N* (%)	10 (29,4)	5 (19,2)	n.s.
Polypnea, *N* (%)	2 (5,9)	2 (7,7)	n.s.
Myalgia, *N* (%)	4 (11,8)	3 (11,5)	n.s.
Desaturation, *N* (%)	0 (0)	0 (0)	
Abdominal pain, *N* (%)	0 (0)	1 (3,9)	n.s.
Diarrhea, *N* (%)	0 (0)	0 (0)	
Change smell, *N* (%)	2 (5,9)	2 (7,7)	n.s.
Change taste, *N* (%)	2 (5,9)	0 (0)	n.s.

ARDS, acute respiratory distress syndrome; BMI; body mass index; IR, insulin resistance; T2DM, type 2 diabetes mellitus; COPD, chronic obstructive pulmonary disease; CKD, chronic kidney disease; Afib; atrial fibrillation; CHD; coronary heart disease; NAFLD; non-alcoholic fatty liver disease; N, number of patients; %, percentage; SD, standard deviation. Chi-square test; ****p < 0.0001, ***p < 0.0002, **p < 0.0021, and *p < 0.0332.

**Figure 1:**
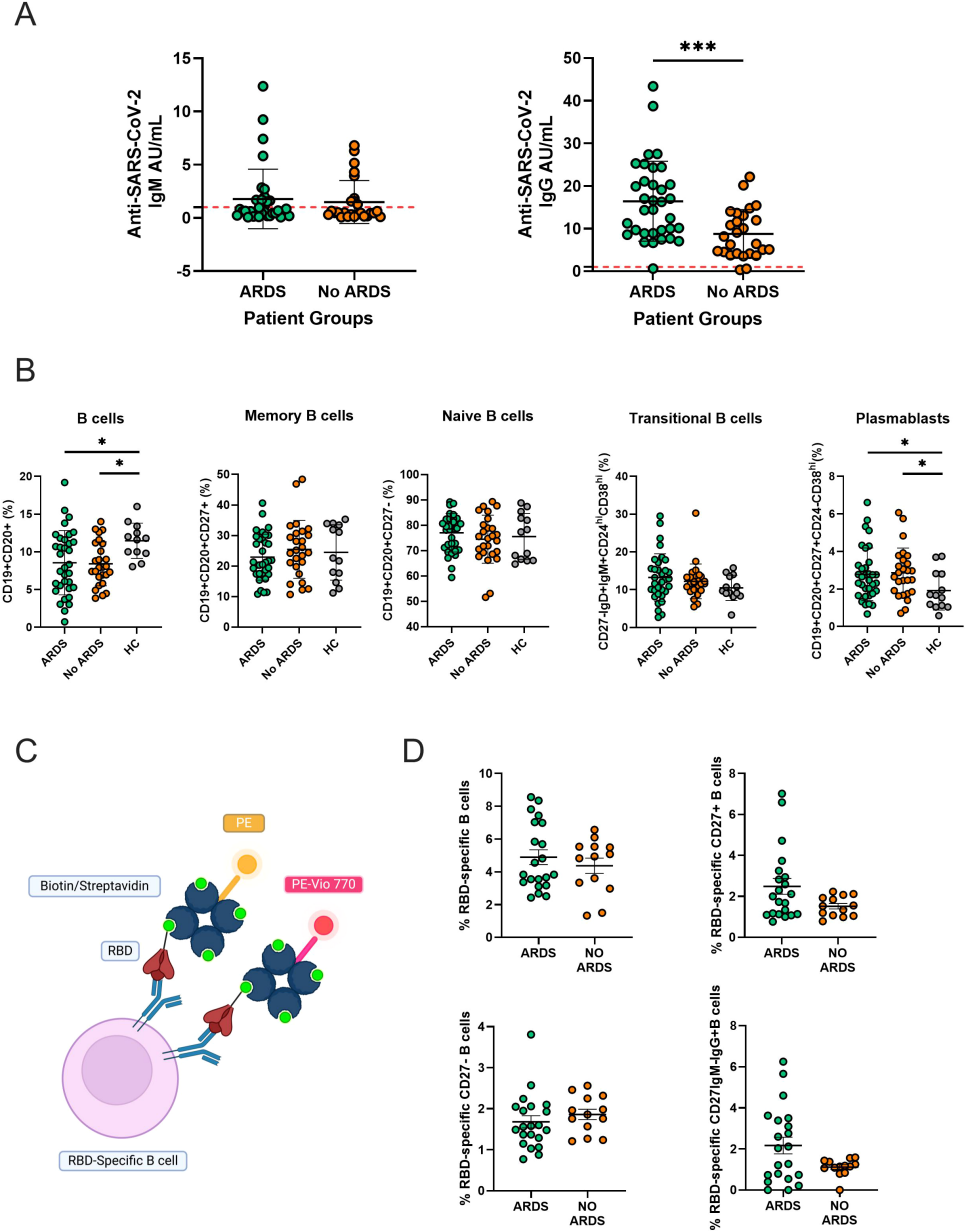
Profile of Anti-SARS-CoV-2 IgM and IgG antibodies and the distribution of B cell subpopulations in patients with COVID-19–4 months post-infection. **(A)** Serum levels of anti-SARS-CoV-2 IgM and IgG antibodies in patients with ARDS (n=34) or without ARDS (n=26). The red dashed horizontal lines indicate the positivity threshold. Statistical significance is indicated with asterisks (***p < 0.001, Mann-Whitney test). **(B)** Percentage of different circulating B cell subpopulations in patients with ARDS, patients without ARDS and healthy controls (HC, n=15). The percentages of total B cells (CD19+CD20+), memory B cells (CD19+CD20+CD27+), naive B cells (CD19^+^CD20^+^CD27^-^), transitional B cells (CD27^-^IgM^+^IgD^+^CD24^hi^CD38^hi^), and plasmablasts (CD19^+^CD20^+^CD27^+^CD24^-^CD38^hi^) are shown. Statistical significance is indicated with asterisks (***p < 0.005, *p < 0.05, Kruskal-Wallis multiple comparisons test) **(C)** Illustrative scheme of the kit used to recognize B cells specific for the receptor-binding domain (RBD) of SARS-CoV-2 using a biotinylated RBD antigen detected with streptavidin conjugated to two fluorochromes, PE-Vivo 770 and PE. Figure by *Biorender.***(D)** Percentage of anti-SARS-CoV-2 specific RBD B cell subpopulation in patients with ARDS (n=21) and patients without ARDS (n=13). The percentages of RBD-specific B cells within total B cells, memory B cells (CD27^+^), naive B cells (CD27^-^), and isotype-switched memory B cells (CD27^+^IgM^-^IgG^-^) are shown. Bars represent the median and interquartile range. The statistics were performed using the Mann-Whitney test.

Several studies investigating antibody production during SARS-CoV-2 infection have reported that IgG levels vary depending on the severity of symptoms during the acute phase, with higher IgG titers being associated with more severe disease. Notably, most of these studies have been conducted in Chinese, Indian, or European populations, often excluding Latin American cohorts (21–23). To assess the humoral response in our cohort, anti-SARS-CoV-2 IgM and IgG levels were measured using CLIA. Our results indicated that analysis of antibody levels in patients with COVID-19 showed no significant differences in IgM levels. However, IgG levels were significantly higher in patients with ARDS compared to those without ARDS (**Figure 1A**). Additionally, circulating B cell populations were analyzed using flow cytometry (**Supplementary Figure S1**). COVID-19 patients, both with and without ARDS, displayed a significantly lower CD19^+^CD20^+^ B cell population compared to healthy controls. No significant differences were observed in memory, naive, or transitional B cell populations between groups. However, the percentage of plasmablasts was significantly higher in both groups of COVID-19 patients compared to healthy donors (**Figure 1B**). Then, RBD-specific B cell subsets were analyzed to evaluate differences in the antigen specific memory response, in which B cells capable of binding RBD-tetramers are considered antigen-specific B cells (**Figure 1C**). Our results did not show a significant difference between groups regarding the different RBD-specific B cell populations analyzed (**Figure 1D**).

### 3.2 Circulating Chemokines CXCL9 and CXCL10 in serum correlates with IgG anti SARSCOV-2 at 4 months post-infection

**Figure 2:**
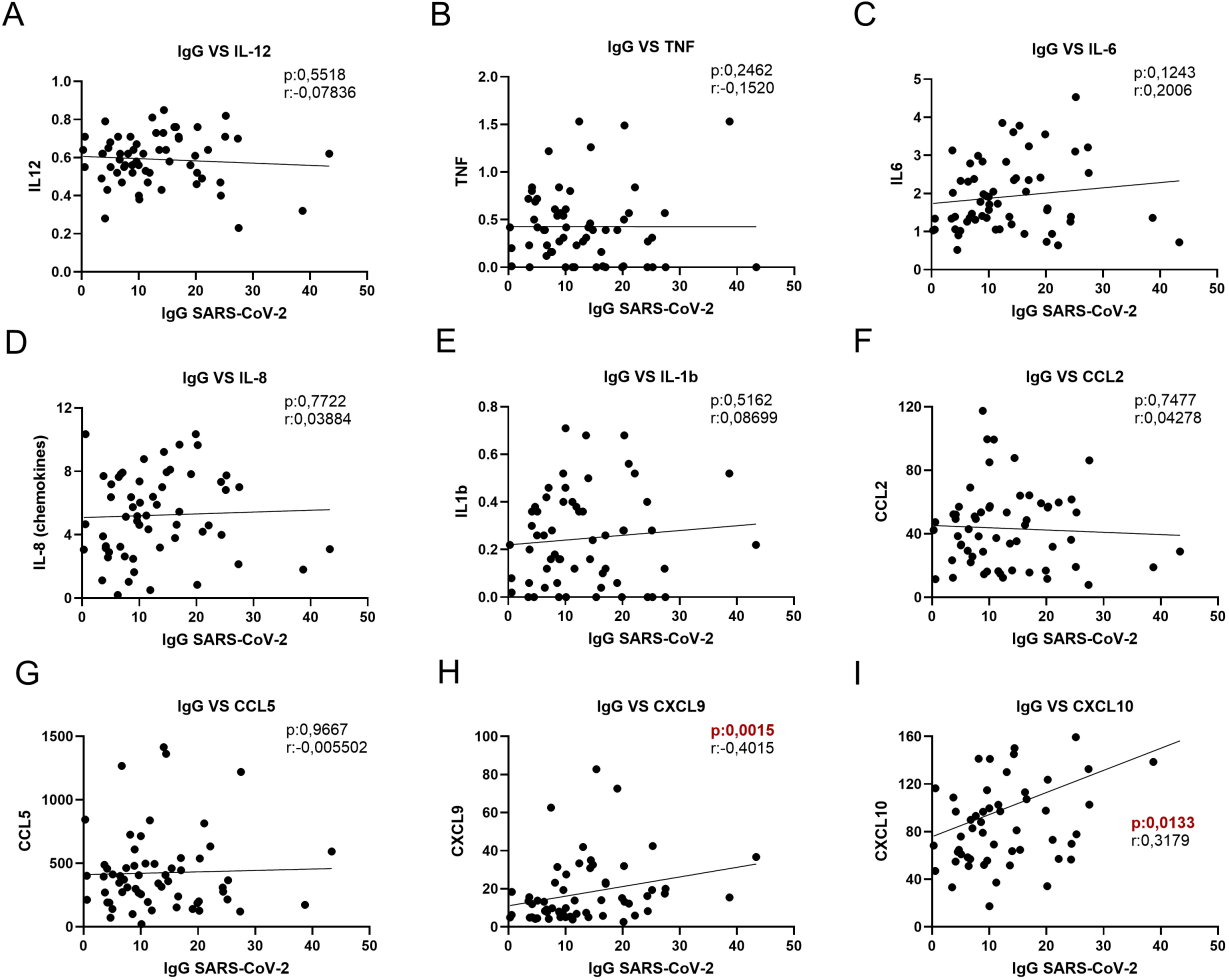
Correlations between anti-SARS-CoV-2 IgG levels and serum cytokine and chemokine levels. **(A)** IgG vs. IL-12, **(B)** IgG vs. Tumor Necrosis Factor (TNF), **(C)** IgG vs. (IL-6), **(D)** IgG vs. IL-8, **(E)** IgG vs. IL-1β, **(F)** IgG vs. CCL2, **(G)** IgG vs. CCL5, **(H)** IgG vs. CXCL9, and **(I)** IgG vs. CXCL10. Each graph shows a linear regression line, the p-value of the correlation and the Pearson or Spearman correlation coefficient (r), according to the normality test. Significant p-values (p < 0.05) are highlighted in red.

Mortality in patients with severe COVID-19 has been linked to the presence of the virus-induced “cytokine storm”. Excessive production of proinflammatory cytokines and chemokines leads to worsening ARDS and widespread tissue damage, resulting in multiorgan failure and increasing the risk of death (24–26). Because increases in these soluble proinflammatory factors exacerbate the immune response, we evaluated the relationship between circulating cytokine and chemokine concentrations and anti-SARS-CoV-2 IgG levels to explore their potential contribution to the humoral immune response. A correlation analysis between the cytokines IL-12 (**Figure 2A**), TNF (**Figure 2B**), IL-6 (**Figure 2C**), IL-8 (**Figure 2D**), and IL-1b (**Figure 2E**) with anti-SARS-CoV-2 IgG levels was evaluated, and the results indicated no significant correlation for any of these cytokines. In contrast, when analyzing chemokines such as CCL2 (**Figure 2F**), CCL5 (**Figure 2G**), CXCL9 (**Figure 2H**), and CXCL10 (**Figure 2I**), we found that only CXCL9 and CXCL10 levels exhibited a significant positive correlation with circulating IgG concentrations.

CXCL9 and CXCL10 bind the CXCR3 receptor and are critically involved in the recruitment of immune cells to sites of inflammation (27, 28). These chemokines have been widely implicated in the pathophysiology of numerous inflammatory and autoimmune conditions, such as systemic lupus erythematosus, multiple sclerosis, and rheumatoid arthritis, where their overexpression is associated with exacerbated tissue damage and poor clinical outcomes (29–32). Additionally, both chemokines have been reported to play a role in the regulation of adaptive immune responses, including the modulation of B cell activity and germinal center dynamics (33, 34).

In the context of viral infections, elevated levels of CXCL9 and CXCL10 have been linked to hyperinflammatory states and disease severity, particularly in respiratory infections such as influenza and COVID-19. Several studies have demonstrated that these chemokines are markedly increased in the serum of patients with severe COVID-19 and correlate with worse clinical outcomes, including the development of ARDS and progression to multi-organ failure (35, 36).

Based on these observations, we hypothesized that CXCL9 and CXCL10 may influence humoral immunity by acting directly or indirectly on B cells. To explore this, we next investigated whether B cells express the CXCR3 receptor, which would allow them to respond to CXCL9 and CXCL10 signaling and potentially contribute to enhanced antibody production.

### 3.3 CXCR3, receptor of chemokines CXCL9 and CXCL10, is express in different subsets of B cells

CXCR3 (C-X-C motif chemokine receptor 3) is a G protein-coupled chemokine receptor spanning seven transmembrane domains and is also the receptor for the chemotactic factors CXCL4 and CXCL11. CXCR3 exists in three known isoforms: CXCR3-A, CXCR3-B, and CXCR3-alt. Among them, CXCR3-A is the most abundantly expressed isoform on immune cells where it plays a critical role in promoting chemotaxis, cell proliferation, survival, and tissue invasion in response to inflammatory stimuli. CXCR3-B is expressed predominantly on non-immune cells, it has been associated with growth inhibition, induction of apoptosis, and anti-angiogenic activity, and it is a unique receptor for CXCL4, but also acts as a receptor for CXCL9, CXCL10, and CXCL11. The CXCR3-alt variant is a truncated isoform containing only four transmembrane domains, is selectively activated by CXCL11 and its biological role remains less well defined (37, 38).

The functional relevance of CXCR3 has been extensively characterized in T lymphocytes, particularly within the pathophysiological context of autoimmune disorders, infections and oncological diseases, where its expression facilitates the targeted migration of effector T cells to sites of inflammation and tumor-associated microenvironments (39–42). In contrast, the expression profile and immunological role of CXCR3 in B lymphocytes remain less well characterized. To date, most studies have focused on the expression of CXCR3 in memory B cells, with evidence suggesting a role in their tissue localization (43, 44). However, investigations into CXCR3 expression in plasma cells are limited, and its biological role in B cell activation, differentiation, or antibody production remains largely unexplored.

**Figure 3:**
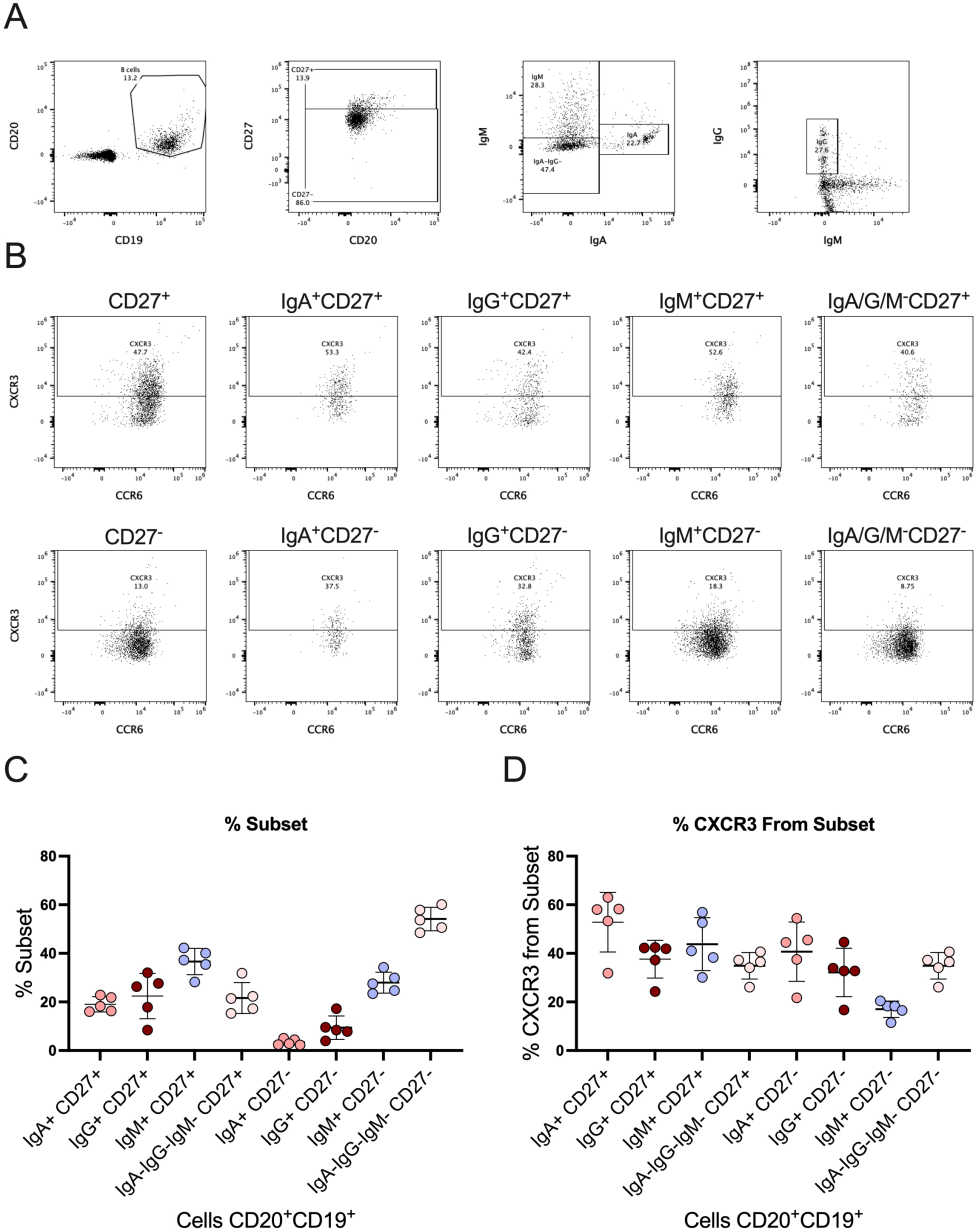
CXCR3 and its expression in different B cell subpopulations. **(A)** Gating strategy for the identification of B cells (CD19^+^CD20^+^) and their subpopulations CD27^+^, CD27^-^, IgM^+^, IgA^+^, IgG^+^. **(B)** Gating strategy to identify the percentage of CXCR3 expression in different subpopulations of B cells. **(C)** Percentage of different B cell subpopulations within the total B cell population (CD19^+^CD20^+^). Each point represents an individual, and the bars represent the median and interquartile range. **(D)** Percentage of CXCR3 expression within the different B cell subpopulations. Each point represents an individual, and the bars represent the median and interquartile range.

To address this, we investigated CXCR3 expression in different B cell subpopulations using spectral flow cytometry (**Figures 3A, B**). We first identified the distribution of B cell populations (**Figure 3C**) and then analyzed CXCR3 expression in each subpopulation (**Figure 3D**). Our results demonstrated the presence of CXCR3-positive populations in all B cell subpopulations, suggesting that these cells might respond to CXCL9 and CXCL10 signaling. These findings highlight a potential pathway through which proinflammatory chemokines could directly influence B cell function and antibody production, thereby contributing to the dysregulated humoral response observed in severe COVID-19 and other inflammatory diseases.

### 3.4 Chemokine CXCL9 promotes B cell activation *in vitro*

To evaluate the modulatory effects of the chemokines CXCL9 and CXCL10 on B cell activation and differentiation into antibody-producing plasma cells, a three-phase culture protocol was optimized based on previously published protocols for *in vitro* B cell activation and differentiation (45, 46).

**Figure 4:**
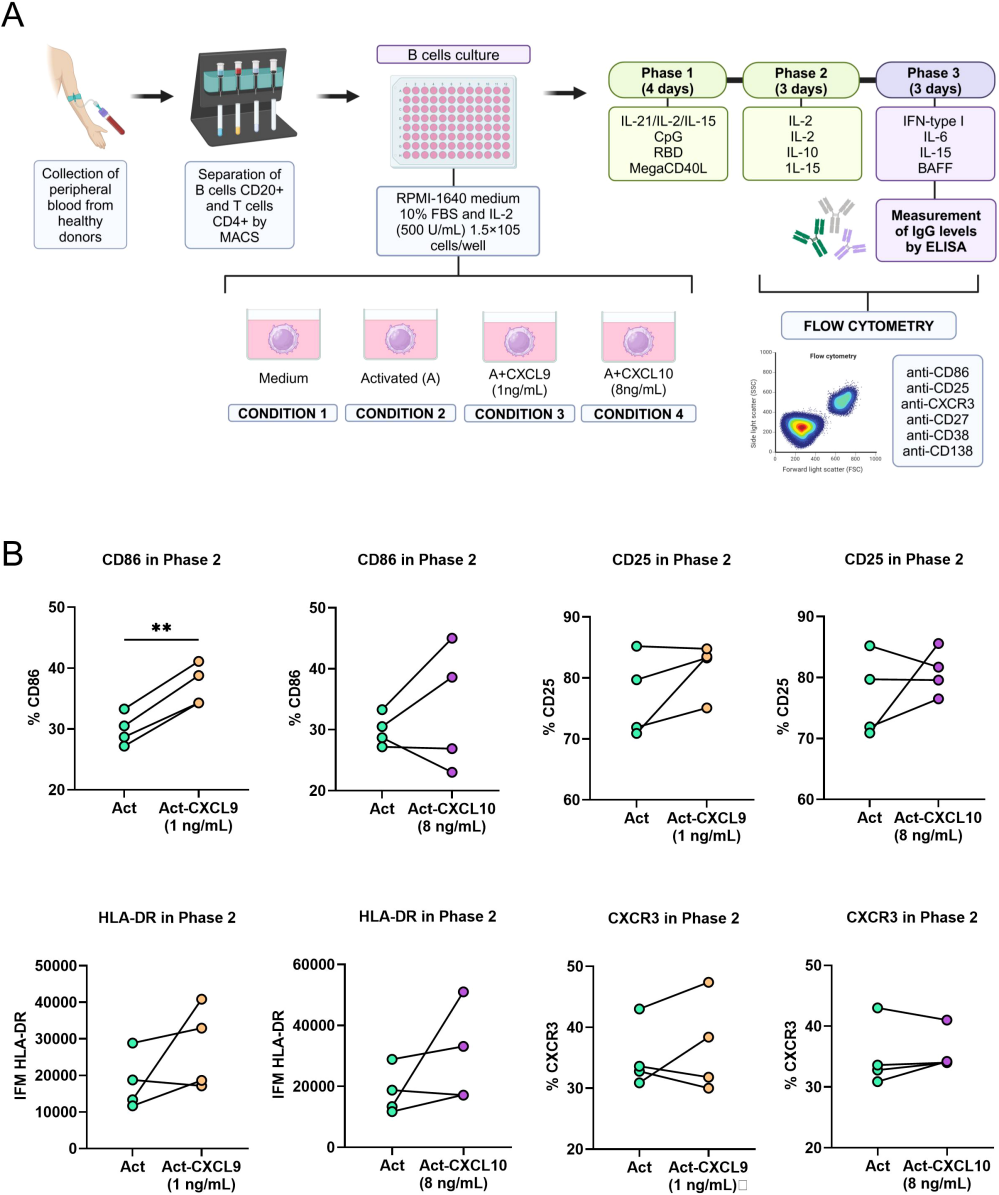
Analysis of activation markers in response to chemokines CXCL9 and CXCL10 during *in vitro* activation of B lymphocytes. **(A)** Schematic of the experimental protocol for the three-phase *in vitro* culture for the activation and differentiation of B cells. **(B)** Paired graphs comparing the expression of different markers in Phase 2 between activated B cells (Act) and activated B cells treated with CXCL9 (1 ng/mL) or CXCL10 (8 ng/mL). The percentage of expression for CD86 and CD25, the mean fluorescence intensity (MFI) for HLA-DR, and the percentages of CXCR3-positive cells are shown. Each line connects the corresponding data from the same donor. Statistics were analyzed using the paired t test or the Wilcoxon test depending on the normality of the data. Statistical significance is indicated by asterisks (**p < 0.01). Data correspond to *n* = 4 donors (2 male, 2 female).

Our culture system comprises two sequential activation stages, during which B cells are exposed to a defined cocktail of cytokines and co-stimulatory molecules, followed by a final differentiation phase. The protocol included four conditions: medium only (negative control), activation (positive control), activation with the addition of CXCL9 (A + CXCL9), and activation with the addition of CXCL10 (A + CXCL10). B cell activation and differentiation were assessed by flow cytometry and confirmed by quantifying total IgG production via ELISA (**Figure 4A**).

Flow cytometric analysis demonstrated that the presence of CXCL9 significantly enhanced CD86 expression compared with the activated control lacking chemokines, whereas CXCL10 had no detectable effect on CD86 levels (**Figure 4B**). In contrast, surface expression of CD25, HLA-DR and CXCR3 remained comparable across all experimental groups following the completion of phase 2.

### 3.5 Chemokines CXCL9 and CXCL10 induce B cell differentiation and IgG secretion *in vitro*

**Figure 5:**
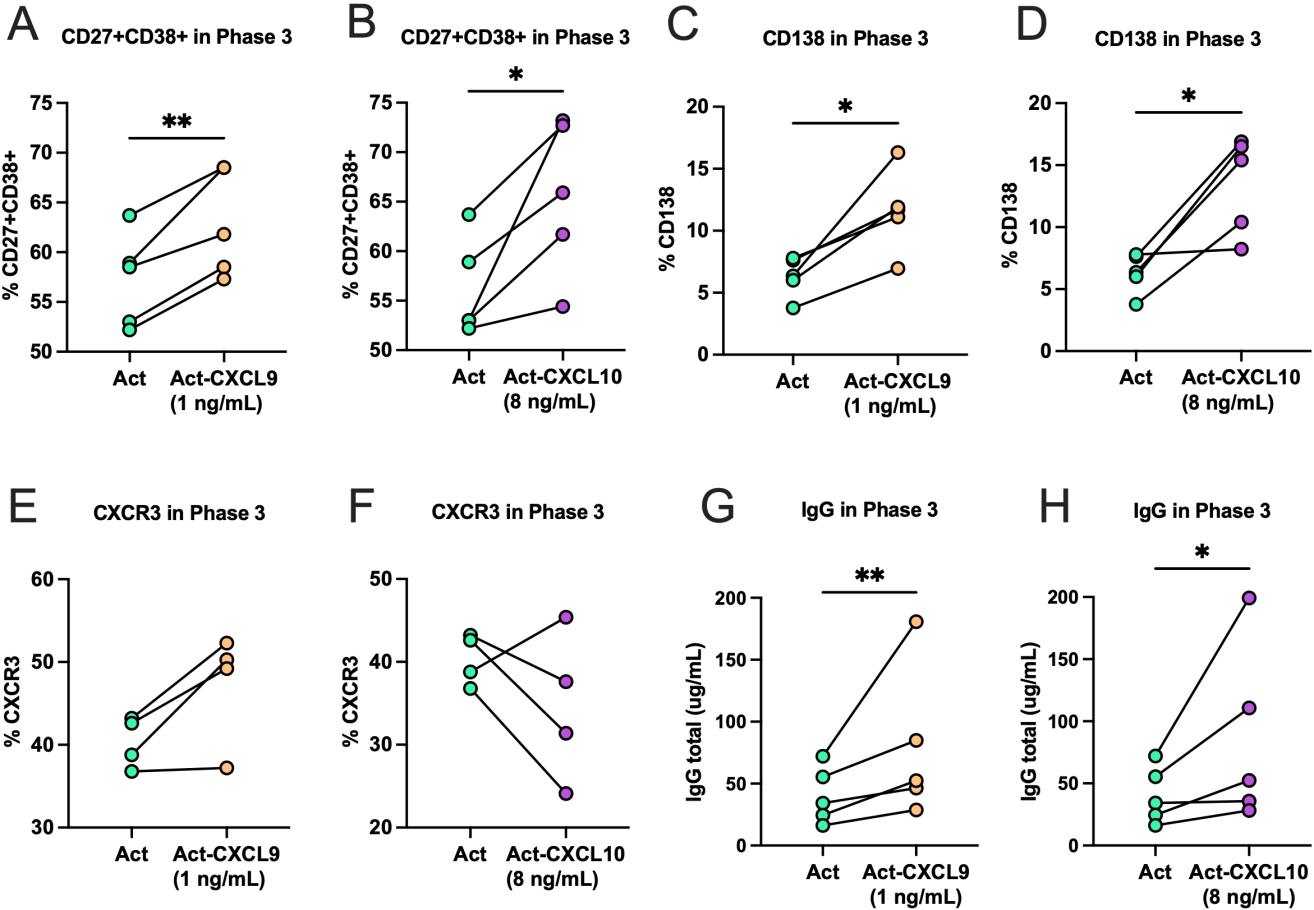
Effect of CXCL9 and CXCL10 on differentiation and IgG production by B cells in Phase 3 culture. **(A)** Percentage of expression for CD27^+^CD38^+^ between activated B cells (Act) and activated B cells treated with CXCL9 (1 ng/mL) or **(B)** CXCL10 (8 ng/mL). **(C)** Percentage of expression for CD138 between activated B cells (Act) and activated B cells treated with CXCL9 or **(D)** CXCL10. **(E)** Percentage of expression for CXCR3 between activated B cells (Act) and activated B cells treated with CXCL9 or **(F)** CXCL10. **(G)** Total concentration of IgG (µg/mL) secreted into the culture medium compared after activation and activation with CXCL9 or **(H)** CXCL10. Each line connects the corresponding data from the same donor. Statistics were analyzed using the paired t test or the Wilcoxon test depending on the normality of the data. Statistical significance is indicated by asterisks (*p< 0.05, **p < 0.01). Data correspond to *n* = 4 donors (2 male, 2 female).

At the end of phase 3, plasma cell differentiation was evaluated by flow cytometric analysis of CD27^+^CD38^+^ and CD138 subsets, CXCR3 surface expression, and quantification of total IgG antibody in culture supernatants by ELISA. Our results indicate that CD27^+^CD38^+^ cells significantly increase in the presence of CXCL9 and CXCL10 compared to activated control (no chemokines) (**Figures 5A, B**). The plasma cell marker CD138 also increased significantly higher in the conditions with chemokines compared to the control (**Figures 5C, D**). In contrast, CXCR3 expression on B cells remained consistent across all conditions (**Figures 5E, F**). Consistent with these phenotypic changes, total IgG production was significantly higher in CXCL9 and CXCL10 treated cultures than in activated controls (**Figures 5G, H**), indicating that both chemokines enhance *in vitro* differentiation of B cells into antibody-secreting plasma cells.

### 3.6 Chemokine CXCL9 upregulates CD40L and CXCR3 in CD4^+^T cells

CD4^+^ T cell activation is an essential step in T cell-dependent B cell activation. This process requires CD40L on activated CD4^+^ T cells to interact with CD40 on B cells, which drives germinal center formation, isotype switching, somatic hypermutation, the generation of long-lived plasma cells and memory B cells. CD40 signaling is also essential for germinal center B cell survival, and its dysregulation results in various autoimmune diseases (47–50). In this context, we investigated how inflammatory mediators, such as the chemokines CXCL9 and CXCL10, influence CD40L expression, seeking to reveal novel mechanisms that control T cell-dependent B cell responses.

**Figure 6:**
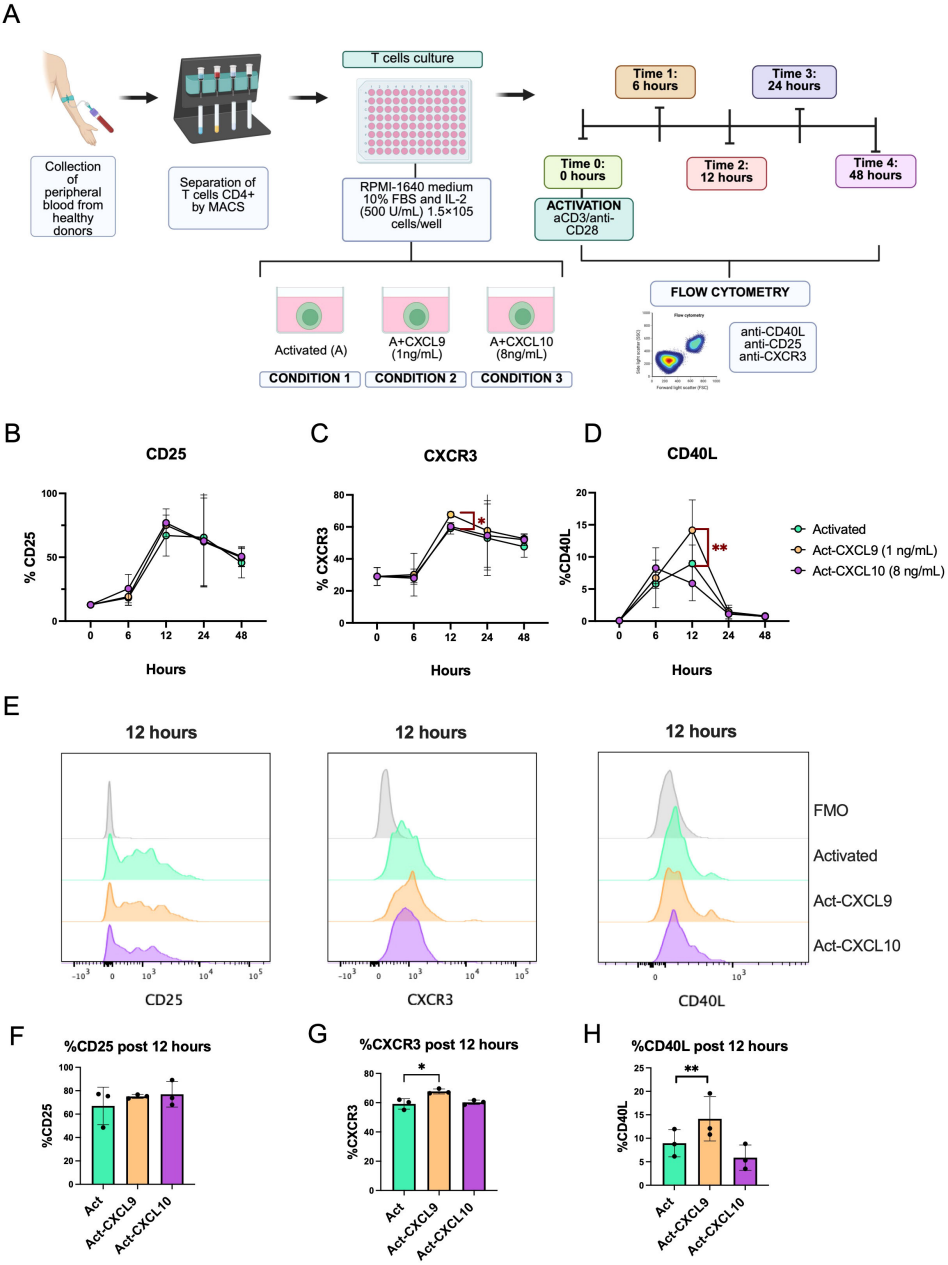
Kinetics of expression of activation markers and CXCR3 receptor expression in CD4^+^ T cells stimulated with CXCL9 and CXCL10 *in vitro.***(A)** Schematic of the experimental protocol for CD4^+^ T cell activation *in vitro.***(B)** Kinetics of CD25 activation marker expression on CD4^+^ T cells over time in the activated condition and activated with CXCL9 or CXCL10. **(C)** Kinetics of CXCR3 chemokine receptor expression on CD4^+^ T cells over time under the different stimulation conditions. **(D)** Kinetics of CD40 ligand (CD40L) expression on CD4+ T cells over time under the different stimulation conditions. **(E)** Flow cytometry histograms of CD25, CXCR3, and CD40L expression on CD4^+^ T cells 12 hours after activation under different culture conditions. **(F)** Bar graphs of the percentage of CD25, **(G)** CXCR3, and **(H)** CD40L expression at 12 hours post-activation under different culture conditions. The points represent the mean and the error bars the standard deviation. Statistical significance is indicated (*p < 0.05; **p < 0.01, two-way ANOVA followed by multiple comparisons). Data correspond to *n* = 5 donors (3 male, 2 female).

To determine whether the IFN-γ–inducible chemokines CXCL9 and CXCL10 modulate T cell help, purified CD4^+^ T cells were stimulated with anti-CD3/CD28 beads under three conditions: beads alone (Activaded), beads + CXCL9 (Act + CXCL9), or beads + CXCL10 (Act + CXCL10)—and harvested at 0, 6, 12, and 24 h. Surface expression of CD25, CXCR3, and CD40L was quantified by flow cytometry (**Figure 6A**). Our results show that CD25 expression increases over time, showing a peak at 12 hours after activation and then decreasing again with no differences observed between conditions (**Figure 6B**). Something very similar is observed in the expression of CXCR3, where expression is stable from 0 to 6 hours, then a peak is observed at 12 hours and then decreases. Statistically significant differences in CXCR3 expression were observed at 12 hours between the control activated only by beads and the condition activated with the addition of CXCL9, where expression was higher in chemokine condition (**Figure 6C**). CD40L expression starts from basal levels from 0 to 6 hours to show a peak at 12 hours where CD40L expression is significantly higher in the condition with CXCL9 compared to the control activated only by beads. Then after 24 hours, expression in all conditions decreases again to basal levels in all conditions (**Figure 6D**). **Figures 6E-H** best illustrate the differences observed between the markers, primarily CXCR3 and CD40L, at 12 hours, the time point at which significant differences were observed. These findings suggest that CXCL9 selectively increases the overexpression of CXCR3 and CD40L on activated CD4^+^ T cells, identifying a possible mechanism by which CXCL9 enhances T cell-dependent B cell activation and subsequent humoral immunity.

### 3.7 CXCL9 and CXCL10 chemokine levels and IgG levels remain elevated in patients with pulmonary sequelae 4 months after COVID-19 infection.

**Figure 7:**
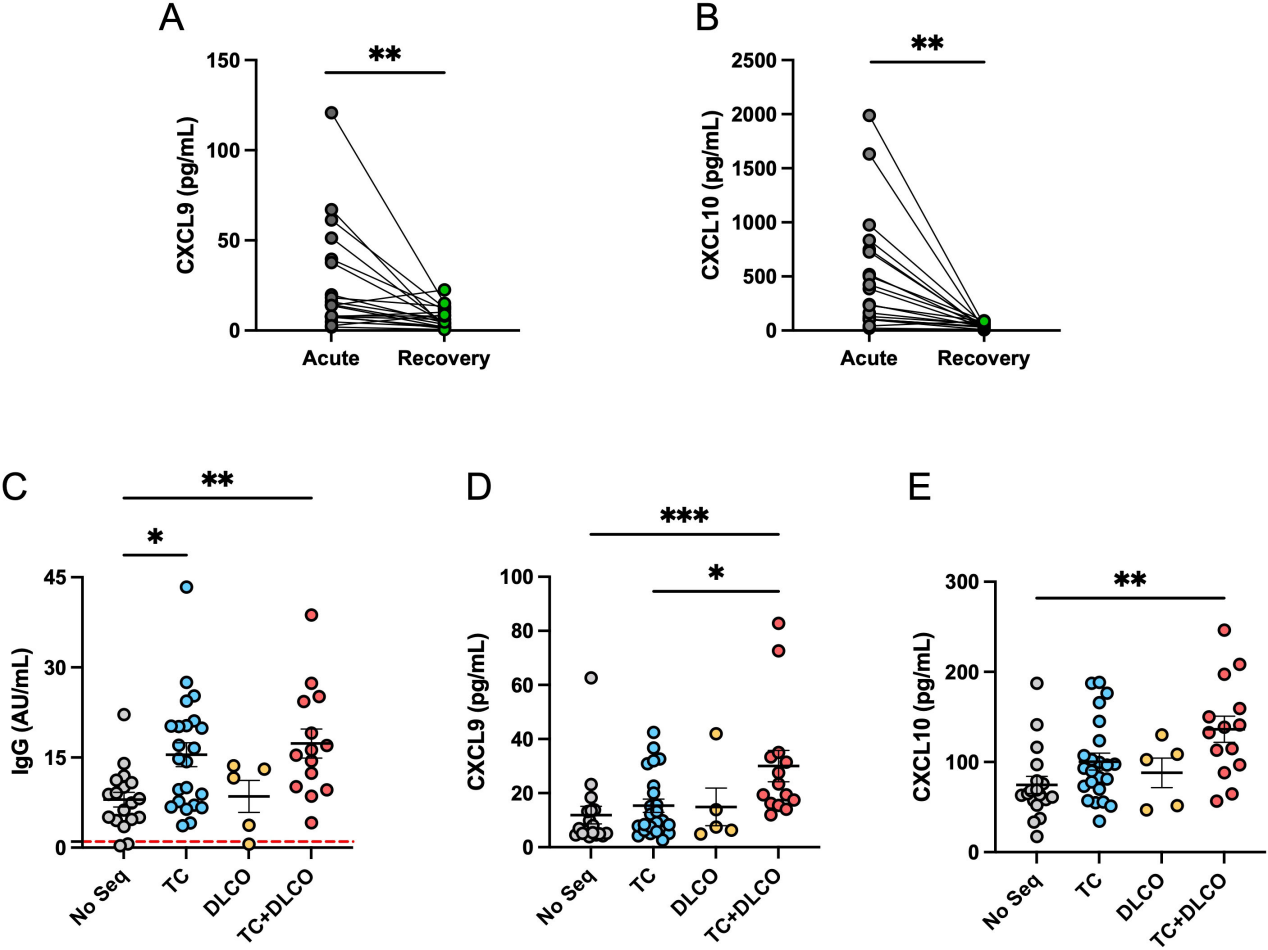
CXCL9 and CXCL10 levels in the acute and recovery phases and their association with post-acute pulmonary sequelae. **(A)** Comparison between serum levels of CXCL9 and **(B)** CXCL10 in patients with ARDS during the acute phase and the recovery phase (4 months after COVID-19). **(C)** Anti-SARS-CoV-2 IgG levels (AU/ml) in patients without sequelae (No Seq, n=18), with altered computed tomography (CT) (n=22), with reduced carbon monoxide diffusing capacity (DLCO, n=5), and with both conditions (CT+DLCO, n=14). The red dashed horizontal lines indicate the threshold for positivity. **(D)** CXCL9 levels (pg/mL) and **(E)** CXCL10 levels (pg/mL) in the same groups of patients described in **(A)** For **(A, B)**, statistical analysis was performed using the Student t test for paired samples, while for **(C-E)**, the Kruskal-Wallis test was used (*p < 0.05, **p < 0.01, ***p < 0.001).

Because cytokine and chemokine levels are relevant in the context of ARDS patients with COVID-19, serum levels of CXCL9 and CXCL10 during the acute phase were compared to both chemokines’ levels during recovery phases (4 months post-infection). A significant increase in CXCL9 and CXCL10 chemokine levels was observed in samples from patients in the acute phase compared to the recovery phase of COVID-19 (**Figures 7A, B**). This data suggests that CXCL9 and CXCL10 participated in the cytokine storm during the acute phase, therefore the increment is occurring during the development of ARDS and the humoral response.

Later in time, pulmonary complications are among the most frequently reported long-term sequelae following SARS-CoV-2 infection. Radiological abnormalities on chest computed tomography (CT) scans, such as ground-glass opacities and fibrotic-like changes, have been commonly documented during follow-up, particularly in patients who required invasive mechanical ventilation during the acute phase. Additionally, reduced diffusion capacity for carbon monoxide (DLCO) is a well-established marker of impaired pulmonary function in post-COVID-19 patients (51–54).

Given the importance of persistent inflammation and immune activation in the pathogenesis of post-COVID lung damage, we evaluated whether levels of anti-SARS-CoV-2 IgG and the pro-inflammatory chemokines CXCL9 and CXCL10 were associated with pulmonary sequelae at four months post-infection. Patients were classified into four groups: those without pulmonary sequelae (No Seq), those with radiological abnormalities on CT (abnormal CT), those with reduced DLCO (DLCO) and those with both CT abnormalities and reduced DLCO (abnormal CT + DLCO). Whereas abnormal CT indicated structural damage, reduced DLCO indicated functional damage. Our results show that anti-SARS-CoV-2 IgG levels were significantly elevated in patients with CT abnormalities, and even higher in those with CT abnormalities and reduced DLCO, compared with individuals without pulmonary sequelae (**Figure 7C**). Regarding chemokines, CXCL9 levels were significantly elevated in patients with combined CT and DLCO abnormalities, compared with the groups with CT alone and without sequelae (**Figure 7D**). A very similar pattern was observed for CXCL10, where levels were significantly higher in patients with altered CT and reduced DLCO compared to those without sequelae (**Figure 7E**). Taken together, these findings indicate that elevated levels of anti-SARS-CoV-2 IgG are associated with structural lung damage, without necessarily affecting functional damage, whereas the presence of higher levels of CXCL9, and CXCL10 are only associated with persistent pulmonary sequelae, evidenced in individuals with radiological and functional lung impairment. Since the DLCO measures how quickly oxygen moves from the lungs into the bloodstream, the presence of inflammatory chemokines in circulation could affect this exchange, as previously proposed (53), whereas the exacerbated humoral response affect the structure of the lung by mechanism such as complement-dependent cytotoxicity, antibody-dependent cellular cytotoxicity and immune complex-mediated tissue damage. In combination, a sustained humoral and chemokine-mediated inflammatory responses may contribute to the pathophysiology of long-term pulmonary complications following SARS-CoV-2 infection, even up to 4 months post-acute phase.

## 4 Discussion

The main aim of this study was, first, to provide an analysis of the humoral immune response and B cell dynamics in a cohort of 60 COVID-19 patients who had different severity during the acute phase of infection, and second, to study the role of the chemokines CXCL9 and CXCL10 in the activation, differentiation, and modulation of antibody production. Our results show a significant increase in anti-SARS-CoV-2 IgG antibody levels in patients who developed ARDS compared to those without ARDS, which is consistent with previous findings by other authors indicating an elevation between disease severity and heightened humoral responses in other patient populations (14–16, 55, 56). This suggests that a more robust and potentially sustained IgG response is associated with severe COVID-19. Similar observations have been reported in other lung inflammatory conditions, where excessive or dysregulated IgG responses contribute to immune complex formation, complement activation, and tissue damage. High levels of these immunoglobulins have also been associated with a worse prognosis in patients with interstitial and autoimmune lung diseases, where elevated IgG concentrations were linked to persistent inflammation, pulmonary fibrosis, and reduced lung function (57–59). Conversely, several studies have shown that high IgG titers following vaccination are associated with protection because these antibodies display high affinity and neutralizing capacity, efficiently blocking pathogen entry and correlating with reduced viral load and milder clinical outcomes (60–62). This apparent discrepancy highlights the dual nature of IgG antibodies: protective when generated under controlled antigen exposure and balanced immune activation, but potentially pathogenic when produced during persistent antigen stimulation and an exaggerated inflammatory environment. Together, these findings underscore that the functional outcome of antibody responses depends not only on their magnitude, but also on the quality and context of the immune activation that induces them.

In our setting, at four months post-infection, we observed no significant differences in IgM levels between patients with ARDS and those without. This contrasts with findings from the acute phase, where several studies reported elevated IgM levels in severe cases (63–65). For example, an analysis using electrochemiluminescence assays demonstrated higher early IgM responses in individuals who subsequently developed severe COVID-19 compared to milder cases (65). The discrepancy between these acute-phase observations and our later findings likely reflects the transient nature of the IgM response. Longitudinal studies have consistently shown that IgM levels peak within the first few weeks after symptom onset and decline rapidly (66, 67). In one cohort study, IgM positivity had already fallen to near baseline in most patients by four months post-infection (66). Thus, the lack of elevated IgM in our cohort at this time point is consistent with expected immunoglobulin kinetics.

Analysis of circulating B cell populations using flow cytometry revealed that, following SARS-CoV-2 infection, both groups of COVID-19 patients (with and without ARDS) showed a significantly higher percentage of total B cells (CD19+CD20+) and plasmablasts (CD19+CD20+CD27CD24-CD38^hi^) compared to healthy controls. This increase highlights sustained B cell activation and differentiation response, which contributes to the elevated IgG levels observed in ARDS patients. Of note, while the percentages of naive, memory, and transitional B cell subsets remained comparable to those of controls, the selective increase in plasmablasts suggests a transformation of post-acute B cell subpopulations toward antibody-secreting phenotypes, while other B cell subsets may require additional monitoring and analysis to observe possible changes. This prolonged presence of plasmablasts is consistent with immunophenotypic studies demonstrating prolonged plasmablast activation during convalescence, potentially supporting continued humoral immunity beyond the acute phase of disease (68). Furthermore, immunophenotyping of convalescent COVID-19 patients by Ryan FJ et al. (2022) revealed sustained alterations in adaptive immune subsets—including helper, follicular, and regulatory T cells—accompanied by durable anti-Spike and anti-receptor binding domain (RBD) IgG responses persisting up to six months post-infection, supporting a state of prolonged B cell lineage activation. These findings suggest that recovery from COVID-19 is characterized by a lasting shift toward plasmablast predominance, while other B cell populations remain relatively stable, highlighting the need for further monitoring of B cell dynamics in the post-acute phase (69).

On the other hand, despite the differences in the levels of IgG and percentages of plasmablasts in circulation, no significant differences were observed in the percentage of RBD-specific B cells between patients with and without ARDS in any of the subpopulations analyzed. It should be noted that antigen-specific B cells represent a low percentage population in peripheral blood (70), especially during convalescence, making their identification at 4 months post-infection notable and indicative that both groups of COVID-19 patients can mount an effective antigen-specific B cell response. Furthermore, this suggests that the magnitude of the B cell response directed at the RBD of the viral spike protein is similar regardless of disease severity at 4 months post-infection. Supporting this, previous studies have shown that RBD-specific memory B cells remain detectable in circulation for at least six months following infection, even as antibody levels decline (71). It is possible that the increased anti-SARS-CoV-2 IgG levels observed in ARDS patients reflect a broader antibody response targeting additional viral epitopes, possibly driven by inflammation-induced polyclonal B cell activation. Further studies are needed to characterize the epitope specificity and functional quality of the antibody response in patients with differing disease severities.

A key finding of this study is the significant correlation between serum levels of the chemokines CXCL9 and CXCL10 and anti-SARS-CoV-2 IgG titers in patients recovering from COVID-19. These chemokines have been extensively implicated in the pathogenesis of severe disease, particularly in the context of the cytokine storm observed during ARDS (35, 72). While CXCL9 and CXCL10 are well known for their roles in T cell chemoattraction via CXCR3 signaling (73), their potential contribution to humoral immune regulation remains poorly characterized.

The observed association with IgG levels raises the possibility that CXCL9 and CXCL10 may influence B cell biology beyond mere chemotaxis. Specifically, these chemokines could play a role in the recruitment of B cells to secondary lymphoid organs or inflamed tissues, where they might promote plasma cell differentiation or support plasma cell survival and sustained antibody production. To explore this hypothesis, we optimized the *in vitro* culture system to assess whether CXCL9 and CXCL10 can directly influence B cell differentiation or immunoglobulin secretion.

The finding that both CXCL9 and CXCL10 increased the percentage of CD38^+^CD27^+^ and CD138^+^ cells supports the idea that both may have a modulatory effect on humoral immunity. In parallel, the elevated production of total IgG in the presence of either chemokine further reinforces its ability to enhance plasma cell production and antibody secretion. Furthermore, CXCL9 was found to upregulate the expression of CD86 on B cells, a key costimulatory molecule involved in antigen presentation and interaction with T cells (74, 75). Simultaneously, CXCL9 also induced increased expression of CD40L and CXCR3 on T cells, suggesting that this chemokine may contribute to crosstalk between B and T cells. Since effective humoral responses require coordinated activation between B and T cells, particularly through CD40-CD40L signaling and chemokine-guided colocalization, these findings imply that CXCL9 (and possibly CXCL10) may act beyond chemoattraction, facilitating cellular interactions essential for B cell activation, differentiation, and sustained antibody production. Further studies will be required to delineate the molecular mechanisms underlying these effects and to determine whether they occur *in vivo* during SARS-CoV-2 infection or other inflammatory settings.

Although our study shows an association between elevated levels of CXCL9/CXCL10 and ARDS, this relationship alone cannot establish that elevated levels of circulating chemokines are capable of directly influencing the cause of this pathology. CXCL9 and CXCL10 are chemokines that have been widely implicated in pulmonary pathology, chronic obstructive pulmonary disease (COPD), other interstitial lung diseases (ILD), as well as pulmonary tuberculosis (TB) and also viral pneumonias, linked to COVID-19-associated ARDS (76–79). Under these conditions, the CXCL9/10–CXCR3 axis is consistently associated with the recruitment of activated T cells and macrophages, type 1 immune polarization, and sustained parenchymal inflammation, suggesting that these chemokines act as general amplifiers of pulmonary inflammation, promoting tissue damage, rather than as specific causal triggers of ARDS. In this context, studies revealing the mechanism of action that these inflammatory factors may exert on the progression of alveolar damage and endothelial dysfunction in ARDS are lacking. Experimental studies are needed to determine whether CXCL9/10–CXCR3 signaling is directly involved in lung injury or whether its upregulation reflects secondary immune activation. These analyses will clarify whether these chemokines act as mediators or simply as biomarkers of inflammation in the context of ARDS.Our findings demonstrate a clear association between post-COVID-19 pulmonary sequelae and the magnitude of the humoral immune response. Patients with structural abnormalities on chest computed tomography (CT), as well as those with combined structural and functional impairments (CT alterations and reduced DLCO), exhibited significantly higher anti-SARS-CoV-2 IgG titers compared to individuals without detectable sequelae. This association raises the possibility that sustained humoral activation is linked to the long-term consequences of pulmonary injury. One possible mechanism is the persistence of chronic, low-grade inflammation within the lungs, maintaining B cell activation and supporting sustained IgG synthesis. Another possibility is the presence of residual viral antigens or RNA in pulmonary tissue, acting as a reservoir for continuous immune stimulation. However, the precise immunological pathways involved remain unclear, and targeted longitudinal studies are needed to assess their biological and clinical significance.

Elevated levels of the chemokines CXCL9 and CXCL10 are directly associated with vascular inflammation and long-term pulmonary dysfunction (REF). During the acute phase of SARS-CoV-2 infection, we observed elevated levels of these circulating inflammatory proteins, suggesting that the initial exacerbation of these chemokines, in combination with other factors, contributes to the development of ARDS and functional sequelae at the pulmonary level (53, 80, 81). Importantly, although CXCL9 and CXCL10 levels decrease during the convalescent phase, they remain significantly higher in patients with pulmonary sequelae compared to those without. This pulmonary functional alteration, observed four months post infection, was identified using a visual criterion through computed tomography (CT) imaging (82) and a functional assessment of the alveolar-capillary barrier via the carbon monoxide diffusion capacity test (DLCO) (83).

Notably, the association between ARDS and structural and functional lung impairment has been documented in non-COVID contexts, including influenza-associated ARDS and sepsis-related ARDS (84–86). These conditions frequently result in fibrotic remodeling, reduced gas exchange capacity, and persistent radiographic abnormalities—patterns consistent with our observations in post-COVID-19 ARDS patients (85). It is also possible that the sustained humoral activation observed in these cases could be, at least in part, orchestrated by the chemokines CXCL9 and CXCL10, given their potential roles in B cell recruitment, activation, and differentiation. This raises the possibility that these molecules may represent therapeutic targets for modulating aberrant or prolonged antibody responses. Nonetheless, further research is required to dissect the precise mechanisms through which these chemokines influence the interplay between pulmonary injury and humoral immunity.

## Data Availability

The original contributions presented in the study are included in the article/**Supplementary Material**. Further inquiries can be directed to the corresponding author.
