# Supplementary material for: CXCL9 and CXCL10 support the exacerbated humoral response in recovered COVID-19 patients who developed acute respiratory distress syndrome by promoting plasma cell differentiation, whereas CXCL9 also induces CD40L and CXCR3 upregulation on T helper cells
Source: Front Immunol. 2025 Dec 4;16:1684704. doi: 10.3389/fimmu.2025.1684704 (PMC12711852; doi:10.3389/fimmu.2025.1684704)
Supplement: Supplementary file 1 [file Table1.docx]

Supplementary Material

#
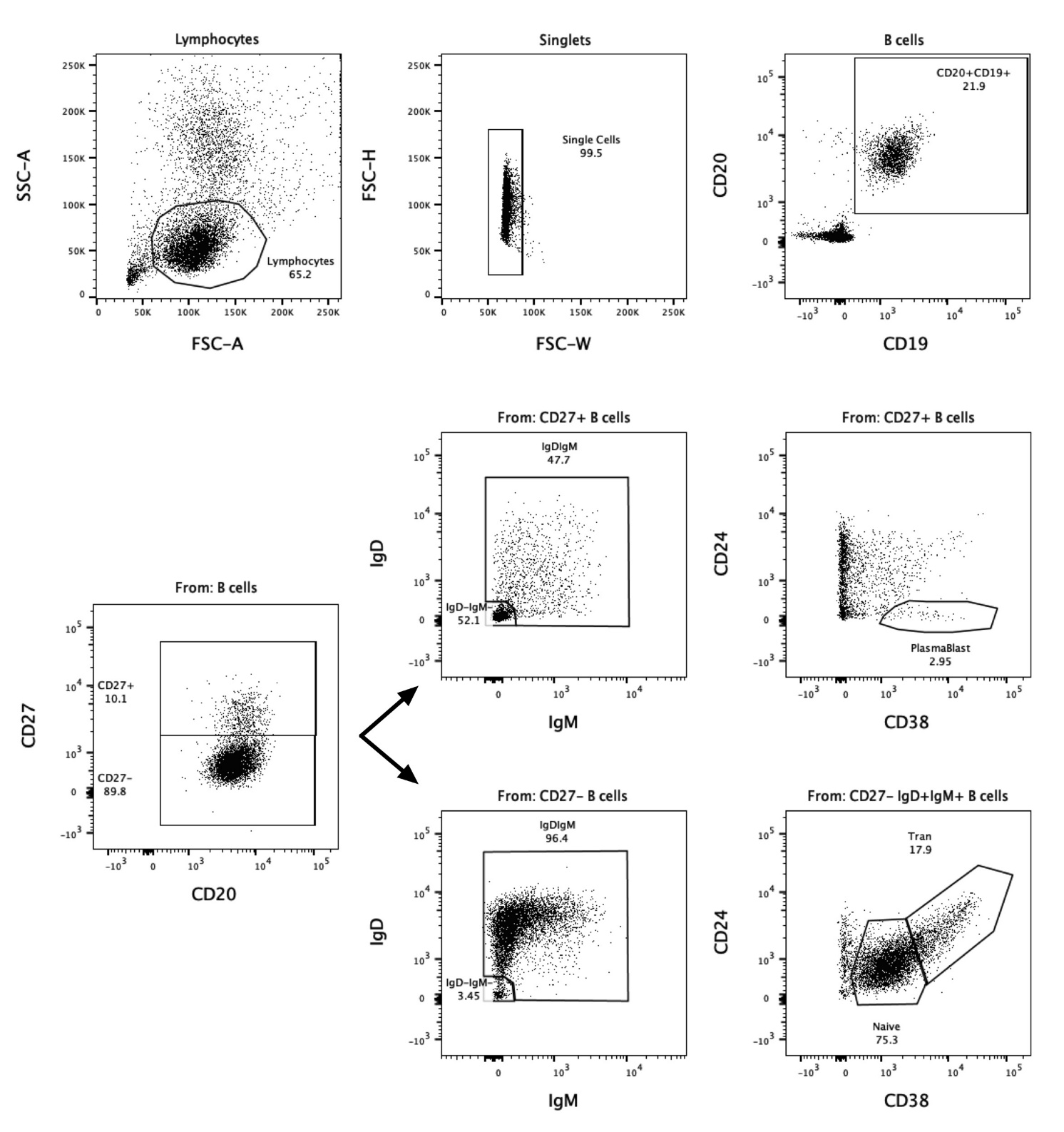
Supplementary Figures

**Supplementary Figure 1. Gating strategy for the identification of peripheral B cell subpopulations by flow cytometry.** Lymphocytes were first gated based on forward scatter area (FSC-A) and side scatter area (SSC-A) parameters. Single cells were selected using FSC-height (FSC-H) versus FSC-width (FSC-W) to exclude doublets. Total B cells were identified as CD19⁺CD20⁺ events. Within the B cell gate, CD27 expression was used to separate memory (CD27⁺) and naive (CD27⁻) populations. From CD27⁺ B cells, IgD and IgM expression distinguished IgD⁺IgM⁺ from switched memory (IgD⁻IgM⁻) subsets, while CD24 versus CD38 expression identified plasmablasts (CD24⁻CD38^hi). From CD27⁻ B cells, IgD and IgM expression separated naive (IgD⁺IgM⁺) from double-negative (IgD⁻IgM⁻) subsets; the naive population was further subdivided into transitional (CD24⁺CD38⁺) and mature naive (CD24^int^CD38^int^) cells. Representative percentages for each population are shown.

# Supplementary Tables

Supplementary Table 1. Description of Healthy Controls characteristics (n=11*)

|  | **Healthy Controls** |
| --- | --- |
| **Gender  Male:Female (%)** | 5:6  (45,5:54,5) |
| **Age (years), (SD)** | 39,5±8,2 |
| **ABO Group** |  |
| A, *N* (%) | 2 (18,2) |
| B, *N* (%) | 0 (0) |
| AB, *N* (%) | 0 (0) |
| O, *N* (%) | 9 (81,8) |
| Weight, Kg (SD) | 78,6±14,1 |
| Height, cm (SD) | 170,9±9,8 |
| BMI, Kg/m^2^ (SD) | 26,9±4,2 |
| Neck circumference, cm (SD) | 36,0±4,0 |
| Waist circumference, cm (SD) | 94,4±11,4 |
| Hip circumference, cm (SD) | 103,3±11,2 |
| **Tabaco status** |  |
| Current, *N* (%) | 3 (27,3) |
| Former, *N* (%) | 0 (0) |
| Never smoker, *N* (%) | 8 (72,7) |
| **Alcohol usage** |  |
| Never, *N* (%) | 2 (18,2) |
| Occasionally, *N* (%) | 8 (72,7) |
| Frequently, *N* (%) | 1 (9,1) |

**Abbreviation list:** BMI: Body mass index, N: number of patients, %: percentage, SD: standard deviation.
******One missing donor*
